# Text mining for disease surveillance in veterinary clinical data: part two, training computers to identify features in clinical text

**DOI:** 10.3389/fvets.2024.1352726

**Published:** 2024-08-22

**Authors:** Heather Davies, Goran Nenadic, Ghada Alfattni, Mercedes Arguello Casteleiro, Noura Al Moubayed, Sean Farrell, Alan D. Radford, P.-J. M. Noble

**Affiliations:** ^1^Institute of Infection, Veterinary and Ecological Sciences, University of Liverpool, Liverpool, United Kingdom; ^2^Department of Computer Science, Manchester University, Manchester, United Kingdom; ^3^Department of Computer Science, Durham University, Durham, United Kingdom

**Keywords:** big data, machine learning, neural language modeling, clinical records, companion animals

## Abstract

In part two of this mini-series, we evaluate the range of machine-learning tools now available for application to veterinary clinical text-mining. These tools will be vital to automate extraction of information from large datasets of veterinary clinical narratives curated by projects such as the Small Animal Veterinary Surveillance Network (SAVSNET) and VetCompass, where volumes of millions of records preclude reading records and the complexities of clinical notes limit usefulness of more “traditional” text-mining approaches. We discuss the application of various machine learning techniques ranging from simple models for identifying words and phrases with similar meanings to expand lexicons for keyword searching, to the use of more complex language models. Specifically, we describe the use of language models for record annotation, unsupervised approaches for identifying topics within large datasets, and discuss more recent developments in the area of generative models (such as ChatGPT). As these models become increasingly complex it is pertinent that researchers and clinicians work together to ensure that the outputs of these models are explainable in order to instill confidence in any conclusions drawn from them.

## 1 Introduction

Natural Language Processing (NLP) is a rapidly growing branch of machine learning aimed at understanding unstructured text using computational methodologies. NLP provides frameworks for computer systems to understand, interpret, and generate human language, which has important implications for applications such as machine translation, text classification, named entity recognition and summarization (1). NLP involves the development of algorithms and computational models to help achieve these challenging goals. However, language does not always follow defined rules but is complex, ambiguous and context-dependent with complications that include regional and dialect differences, emergence of new words, phrases, abbreviations, and colloquialisms.

Within healthcare, NLP is increasingly used to analyze and extract useful information from large volumes of unstructured clinical text data such as electronic health records (EHRs) and clinical notes in an automated process, enhancing the speed and efficiency of clinical decision-making, supporting the detection of disease outbreaks and the ongoing monitoring of disease incidence and prevalence. One study used EHRs from nine hospitals to support an understanding of the outbreak's transmission routes (2). Another area in which NLP has been applied is pharmacovigilance, to capture adverse drug events being reported within clinical narratives to gauge the prevalence and severity and can support the understanding of multi-drug interactions (3, 4).

With the availability of large volumes of veterinary EHRs through frameworks such as SAVSNET and VetCompass, there is a growing need to develop tools and methodologies to fully utilize the rich source of disease information that lies within them (5, 6). As we will describe, machine learning models have the potential to reveal disease syndromic signals within complex textual inputs and have become increasingly accessible even to researchers on modest budgets. This democratization of access to machine learning methods with the attendant potential to screen clinical records at scale has the potential to enhance our understanding of disease patterns in veterinary medicine profoundly.

In the second part of this mini-series, we will discuss the applications and potentials of machine learning methodology to extract valuable insights from unstructured clinical records. We explore how such tools are the building blocks for improving the capabilities of downstream applications such as disease epidemiology and outbreak surveillance. We examine the role of language models, such as bidirectional encoder representations through transformers (BERT) (7) and generative pretrained transformers such as chatGPT (8), Llama (9) (and there are now many more of these to choose from), to extract word meanings to understand the nuances of language and spelling variations within the corpora to better adapt fixed rule-based systems before evaluating them as independent classification tools. By providing an overview of the field's current state, we aim to highlight the pivotal role of machine learning-based text mining in enhancing companion animal care and disease surveillance in veterinary medicine.

## 2 Text-mining veterinary clinical notes using machine learning

### 2.1 Machine-learning for word meaning

Machine-learning (ML) is becoming increasingly important for text analysis. In many cases ML relies on neural networks. These are computational representations or software simulations of biological neural networks wherein virtual neurons (or nodes) in multiple layers, are interconnected. Each node aggregates the value of connections from nodes in the layer above, the mathematical weighting of these connections adjusts how much any given connection contributes to the activation of a node. In the simplest neural network these weightings are adjusted by evaluating training data over many iterations and at each iteration adjusting these weightings until the input to the network leads to the “correct” output. The number of weightings (connections) is sometimes referred to as the number of parameters (10).

In part one of this mini-series we discussed the utility of keyword searches for identifying features of EHRs. Veterinary free-text invariably includes both technical scientific and colloquial language, including non-standard abbreviations and misspellings. As it is difficult to curate a complete list of the different ways in which veterinary professionals will describe the same observation, dictionary development can be time consuming and the use of standardized ontologies may result in a loss of recall. Furthermore, even the most complete dictionaries will require updating due to the ever changing nature of nature language. However, there are some simple machine learning approaches which can be implemented in order to augment these dictionaries. An example of such approaches is word embeddings.

Word embedding involves creating vector representations of words (coordinates for a word in a multidimensional word-space) which encapsulate word meaning, therefore allowing mathematical analysis of text. An efficient method for creating embeddings, word2vec, was developed by Mikolov et al., training a neural network to predict words in sentences based on the words surrounding them using a large corpus (hundreds of thousands to millions) of documents (11). The resultant neural network weightings are effectively a vector (usually 200–300 numbers) representing the words embedding. Words, spellings and abbreviations with similar meanings can be identified due to the mathematical similarity of the vectors representing them. A similar procedure can be used to “embed” sentences and passages of text giving these numerical representations of their overall meaning (12).

This approach has been used to expand a dictionary of dietary supplements as described in clinical notes (13), with the model identifying between one and 12 variants for each supplement, resulting in retrieval of 8.39% more clinical notes on average. Similarly, word2vec was used to identify misspellings of pharmaceutical words in clinical notes (14) and resulted in identification of 150 new terms which were used to create an extended lexicon.

The specific advantage of this approach is that the model is trained on data taken from the target corpus, allowing development of embeddings more representative of the language used within that corpus. However, word2vec models do not capture remote relationships between words and attribute a single vector for words that might have multiple meanings. This limitation is avoided in later approaches such as ELMO (15) and transformer architecture (as described below).

### 2.2 Language models as tools for record annotation

Language models can accurately capture semantic and syntactic structures, which is critical to leverage the rich sources of information that unstructured clinical narratives have within them. Such language models permit flexibility in understanding by capturing patterns and relationships across large volumes of data rather than pre-defined rules; the dynamic nature of language requires an equally malleable system to capture the many ways clinicians can articulate their notes. Rule-based systems are limited to defined inputs where subtle variants in language can add significant complexities to their design and, therefore, can only practically be used for searching for one item at a time such as for single disease scope studies. Rule-based systems also rely heavily on the developers' domain-specific knowledge and manual readings of the records to produce. Neural network-based architectures present in language models, such as convolutional neural networks (CNNs) and recurrent neural networks (RNNs), were a significant innovation away from statistical and rule-based frameworks and introduced the concept of word embeddings, a movement toward capturing rich contextual word representations (16).

Recently, the transformer architecture, which capitalizes on the concept of attention (the relationship between words in phrases/sentences sometimes separated by some distance), has enabled new state-of-the-art performances across many NLP tasks (17). Transformers are the core architecture behind Bidirectional Encoder Representation for Transformers (BERT) (7), Generative Pretraining (GPT) (18), and Language Models for Dialog Applications (LaMDA) (19). A key difference to word2vec embeddings is that these models allow for context-specific representations of words to allow different meanings to be coded differently. For example the word “discharge” may mean fluid leaking from a wound or releasing a patient from hospital and would have the same embedding in word2vec models but is ultimately treated as different entities in BERT models depending on context (7).

Disease coding frameworks, such as the International Classification of Disease (ICD), provide a robust methodology for understanding mortality and morbidity information for research and epidemiology (20). However, disease coding of unstructured clinical notes is challenging and is inherently time-consuming, expensive, and prone to errors (21–24). For these reasons, the concept of automating such a process with language models has been well-explored, with previous research exploring the application of RNNs, CNNS and, more recently, the incorporation of attention mechanisms and the transformer architectures (25–29). The desire for automated disease annotation frameworks to exist within veterinary medicine is no different. Previous works have aimed to apply SNOMED-CT diagnosis labels using bidirectional long-short-term memory networks (BLSTMs), a variant of RNNs, using 112,558 expert annotations from a tertiary referral centre showing promising results (30). Further works capitalized from this integrating a transformer architecture allowing for a hierarchical organization of automated disease codings (31). Language models have also been used to understand veterinarians reasoning behind an antimicrobial administration; here, a BERT model was additionally trained on 15 million clinical notes from the VetCompass Australia corpus (32).

In summary, language models present promise for automating annotations within unstructured EHRs. Their capacity to analyze extensive datasets and discern intricate linguistic relationships empowers these models to enhance annotation precision and speed, facilitating more comprehensive data analysis in disease epidemiology. An exemplification of this potential lies in the development of PetBERT, a substantial language model trained on a corpus exceeding 500 million tokens sourced from the SAVSNET dataset (33). This dataset comprises clinical narratives from diverse veterinary practices across the UK. Through fine-tuning, PetBERT was transformed into a multi-label classifier proficient in automatically coding veterinary clinical EHRs using the International Classification of Diseases 11 framework. Impressively, it achieved F1 scores surpassing 83% across 20 disease codings with minimal annotation requirements. Moreover, they serve as foundational structures for bolstering disease outbreak detection capabilities. Employing this syndromic labeling system, we identified a documented disease outbreak. Comparative analysis between PetBERT's automated identification and the previously employed clinician-assigned point-of-care labeling strategy revealed PetBERT's capability to identify the outbreak up to three weeks earlier. The demonstrated proficiency of PetBERT in automating coding processes within veterinary clinical narratives underscores the transformative potential of language models in augmenting disease surveillance and timely outbreak detection within veterinary medicine.

### 2.3 Unsupervised machine learning

**Figure 1:**
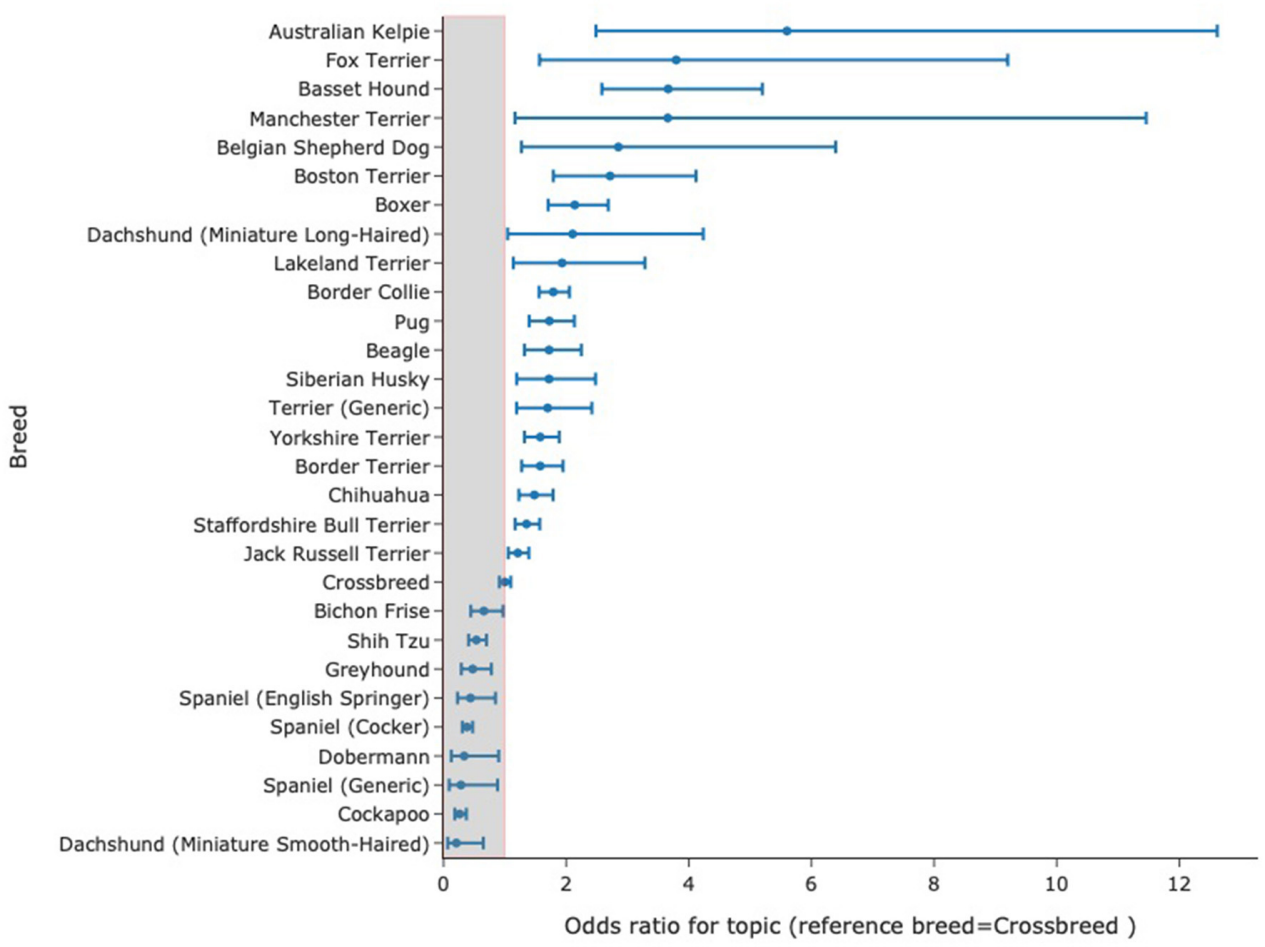
Distribution of odds ratios with 95% confidence interval for a given topic to occur in records from specific breeds. The model comprised 200 topics, generated entirely by the computer without external “hints,” many having clear clinical relevance. Here, odds ratio for records with topic 23 as their most probable topic are calculated against the odds of records from crossbreeds having that topic as the most probable one. Data for topic 23 with key-words: seizure, seizures, bloods, diazepam, epiphen, phenobarb, epilepsy, pexion, lasted, episode.

The machine-learning approaches described above often rely on identifying groups of records for study and in the case of using neural language models will often entail training models using gold-standard annotations made by experts. These are often referred to as 'supervised' systems where the researcher/developer directs what the system learns. Unsupervised systems involve presenting a dataset to a model without stipulating an underlying structure to be detected and to identify the underlying structure within the data in the hope that this will expose relevant clinical features. One such approach is topic modeling. A key approach to this was published by Blei et al. (34) using a statistical approach to text (latent dirichlet allocation) which modeled the assumption that documents contain a distribution of topics and that topics are made up of a specific distribution of words. Reverse engineering this allowed identification of the word distributions present in a set of topics which were inferred from the text itself rather than a prior assumption of what topics were present in the data. This method is made readily available through accessible programming interfaces (35). This approach has been applied to veterinary clinical data allowing clinically relevant topics to be identified in veterinary text to the extent that a topic identified through this method displayed a clear temporal pattern matching a known national outbreak of gastroenteric disease in dogs (36). Topic modeling has been recognized as valuable tool for bioinformatic research (37) and as a tool for clinical research (38, 39).A key feature of topic modeling is that topics discovered in documents are easily interpreted due to each topic having a list of words (and probabilities for those words) that make them up, such that in the example above the outbreak identified above could be clearly seen to be gastroenteric disease given that it comprised words like “diarrhoea,” “vomit,” and “food.” Furthermore, topic-modeling methods allow for evaluation of the weighting of words that comprise the topic with time (or across other categories such as breed, age, and date) highlighting evolution of themes with time that, in the case of disease phenotype, might illustrate emergence of new syndromes (36, 40). More recently, transformer-based models that create whole document embeddings i.e., representation of clinical notes as 768 dimensional arrays based on the meaning of words/tokens in the documents provide another route to topic modeling. Following a reduction of dimensionality in these arrays, a clustering algorithm can be used to cluster documents. An analysis of term-frequency-inverse document frequency (tf-idf) identifies key-words reflected in the these clusters. These words then indicate themes or topics in those documents. This approach is encapsulated in the BERTopic Package (41) and when applied to 1,000,000 SAVSNET records, we produced a topic model comprising 200 different topics each with keywords produced by the model without external prompting or intervention. Each topic is characterized by the keywords present and these usually have a clear clinical correlate for instance words describing ear disease (“ear,” “canal,” “left ear,” “wax,” “right ear,” “otitis,” “discharge,” “left,” “drops,” “both,” “osurnia”) and words describing wound management (“collar,” “wound,” “buster collar,” “poc,” “keep,” “healing,” “looks,” “wound looks,” “healed,” “post,” “off,” “licking,” and “well”). The probability distribution for a given topic could be calculated across breeds. An example of how a topic relating to seizures (with keywords such as seizure, bloods, diazepam epiphen, and epilepsy) created using BERTopic in this way is distributed in records from dogs of different breeds is shown in Figure 1. This data was very similar to published breed-related data on seizuring (42). While topics may not be precise, they can allow rapid and comprehensive screening of large volumes of records for a huge variety of disease phenotypes in a single study.

### 2.4 Generative models

**Figure 2:**
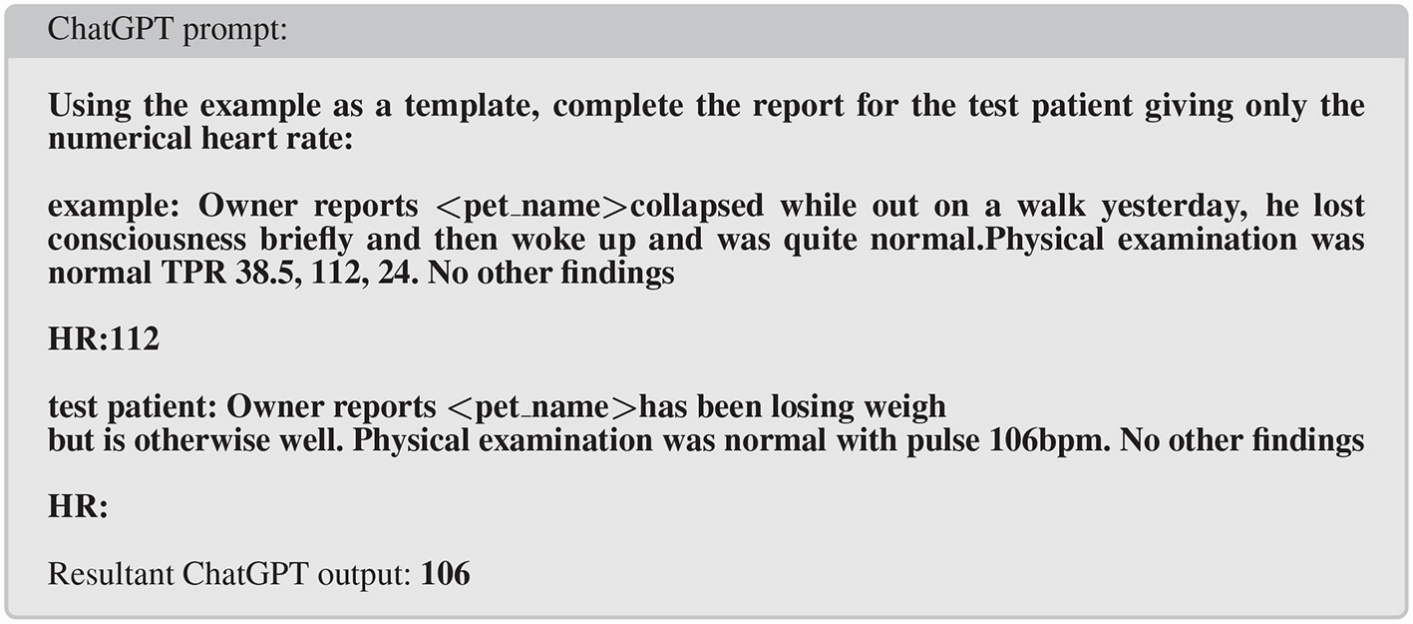
Example of prompt engineering to extract selected data (in this case heart rate) from clinical narratives.

The neural network models described so far have ranged from tens of thousands of connections (parameters) through to hundreds of millions of parameters (in BERT Language models). More recently models have been developed that have billions to trillions of parameters. As a comparison, the human neocortex is estimated to have in the order of 200 trillion synapses (43). These generative models are often trained using tasks such as text completion (particularly next word prediction) and question/answer using massive training sets from diverse sources. Examples include GPT-3, ChatGPT, created by OpenAI (44) and OPT and Llama from Meta (45). The complexity of the internal wiring of these models combined with the extensive training data set leads to behavior that is uncannily human. The main form of interaction is to provide a prompt to which the model generates an answer (this is after all the task the model is trained on). Thus prompting ChatGPT (8) with the text *“tell me in 30 words, why dogs vomit”* returns the text *“Dogs can vomit due to various reasons such as eating too fast, consuming something toxic, having an underlying medical condition, or experiencing motion sickness.”* On the face of it, this conversational dialog appears challenging to extract structured data from but the model will respond with more useful text if prompted in a more structured manner. So called “prompt engineering” allows the prompter to coerce the model to return structured outputs and can be used to classify text from clinical records. For instance when trying to extract an important clinical index of health such as heart rate data from free-text narratives, a suitably engineered prompt can do this (Figure 2). This technique would allow evaluation of a population at scale where previously extensive manual reading or arcane rule-based classification of records might be needed covering only a small subset of available records.

The ability to run prompts across numerous texts is made available through a programming interface which, in theory, could allow for screening of extremely large numbers of records but there are several drawbacks: Firstly, for large volumes, record screening can have a cost per record which can be come substantial for very large datasets (millions of records) with multiple prompts; Secondly, some large language models capture the prompt text for further model training which can lead to some of the potentially sensitive material appearing in outputted text for other users. In order to run such large language models on a local machine (i.e., a copy which will not train on the text or be prompted by external users) requires substantial computing resource. Additionally, when using more complex prompts looking for complicated responses, further issues arise: given the very varied nature of the training data, these models can output opinion that can be misleading, completely fictitious (often referred to as “hallucination”) and even prejudiced/unwholesome. In the case of ChatGPT, beyond the prompt-response training, further fine tuning has been implemented using reinforcement learning from human feedback among other tools to try to forestall misleading or offensive content (44). In our preliminary experiments utilizing ChatGPT to identify overweight animals and body condition scores based on notes written by clinicians during consultations, ChatGPT's performance compared very favorably with a rule-based classifier used for the same task (46). Manual reading of records remains the gold standard against which such approaches are validated.

## 3 Conclusion

Machine learning and artificial intelligence are revolutionizing our ability to automate the generation of signals relating to disease phenotypes in veterinary patients using EHRs. The underlying machinery of the tools is becoming less and less explainable as models with massive numbers of parameters are trained on vast datasets. The impact of large language models will become very significant in the coming years and it will be important that users understand the provenance of data from these models and that researchers work to ensure that the outputs from these models are explainable. While the methods discussed in this second part of the review series have clear benefits in screening huge volumes of data sometimes without requiring any stipulation of the nature of signals to detect, there will remain a role for manual review of records identified by these tools to maintain confidence that valid conclusions are drawn from them.

## Data availability statement

The raw data supporting the conclusions of this article will be made available by the authors, without undue reservation.

## Author contributions

HD: Writing - review & editing, Writing - original draft. GN: Writing - review & editing. GA: Writing - review & editing. MA: Writing - review & editing. NA: Writing - review & editing, Writing - original draft. SF: Writing - review & editing, Writing - original draft. AR: Writing - review & editing. P-JN: Writing - review & editing, Writing - original draft.
